# 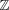-module defects in crystals

**DOI:** 10.1107/S2053273317013882

**Published:** 2017-10-26

**Authors:** Abdullah Sirindil, Marianne Quiquandon, Denis Gratias

**Affiliations:** a Laboratoire de Métallurgie de l’UMR 8247, IRCP Chimie-ParisTech, 11 rue Pierre et Marie Curie, F-75005 Paris, France

**Keywords:** {\bb Z}-modules, intermetallic alloys, defects, twins, dislocations

## Abstract

An analysis is presented of the new types of defects that can appear in crystalline structures where the positions of the atoms and the unit cell belong to the same -module, *i.e.* are irrational projections of an *N* > 3-dimensional (*N*-D) lattice Λ as in the case of quasicrystals. Beyond coherent irrationally oriented twins already discussed in a previous paper [Quiquandon *et al.* (2016) ▸. *Acta Cryst.* A**72**, 55–61], new two-dimensional translational defects are expected, the translation vectors of which, being projections of nodes of Λ, have irrational coordinates with respect to the unit-cell reference frame. Partial dislocations, called here *module dislocations*, are the linear defects bounding these translation faults. A specific case arises when the Burgers vector **B** is the projection of a non-zero vector of Λ that is perpendicular to the physical space. This new kind of dislocation is called a *scalar dislocation* since, because its Burgers vector in physical space is zero, it generates no displacement field and has no interaction with external stress fields and other dislocations.

## Abstract 2

New kinds of defects can appear in crystals where the atoms and unit cell sit on the nodes of 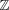-modules, *i.e.* on three-dimensional projections of *N*-dimensional lattices. These defects are the result of the symmetry breaking due to the projection of the structure from *N* to three dimensions. Examples are given that illustrate the processes. A new kind of dislocation, here called a ‘scalar dislocation’, is expected; it generates no deformation and has no interaction with stress fields.

## 1. Introduction   

Many complex intermetallic phases are so-called (periodic) approximants (see, for instance, Gratias *et al.*, 1995 ▸) of quasicrystals (Shechtman *et al.*, 1984 ▸; Shechtman & Blech, 1985 ▸) because their atomic structures are derived from a parent quasicrystal of close composition. This quasicrystal is usually described in the framework of *N*-dimensional (*N*-D) crystallography: the actual structure is generated by cutting an *N*-D periodic object of lattice Λ by the physical three-dimensional space noted 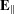, irrationally oriented with respect to the *N*-D periods of Λ (Duneau & Katz, 1985 ▸; Kalugin *et al.*, 1985 ▸; Elser, 1986 ▸).

In that simple scheme, defects are best described in the *N*-D space as locally broken orientational (twins) or translational (boundaries and dislocations) symmetry operations of the *N*-D lattice projected in 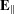. For example, dislocations in quasicrystals (Lubensky *et al.*, 1986 ▸; Socolar *et al.*, 1986 ▸; Wollgarten *et al.*, 1991 ▸, 1992 ▸) are defined using original Volterra constructs in the *N*-D space with Burgers vectors 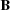 belonging to the *N*-D lattice Λ. For a quasicrystal in a *d*-D space embedded in a 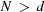-D space, the dislocation line is a manifold of dimension 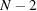 containing the complementary orthogonal space 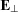 of dimension 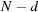 so that the observed dislocation line in 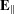 has dimension 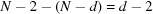, *i.e.* one dimension for three-dimensional objects.

Approximant phases can be described by rational projections of hypothetical quasicrystals defined by *N*-D crystals (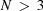) of lattice Λ with atomic surfaces located at rational positions of Λ. This induces the remarkable property that the atomic positions and the unit-cell vectors belong to the same (or its simple submultiples) 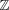-module,^1^ say 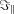, that is the (irrational) projection 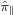 of a lattice Λ in 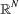 into 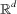 with 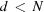: 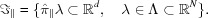



The existence of the 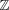-module in crystallography is not confined to quasicrystals and approximants. In fact, several periodic structures have atoms possessing extra non-crystallographic local hidden symmetries which can be viewed as a long-range-ordered decoration on an underlying 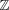-module. Such is the case for the Fe Wyckoff position in the FeAl_3_ phase identified by Black (1955 ▸) and for both Ni and Zr Wyckoff positions in the orthorhombic structure *Cmcm* of NiZr (Kirkpatrick *et al.*, 1962 ▸).

The question addressed in the present paper is the following: what kind of new defects could possibly be generated when the atoms of the crystal, in addition to being periodically spaced, are located on a long-range-ordered subset of the nodes of a 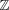-module?

**Figure 1:**
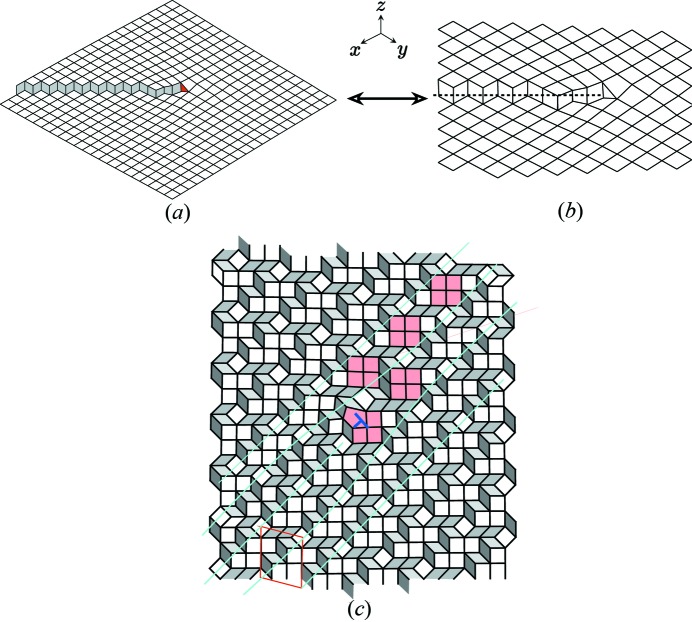
(*a*) A mixed dislocation of Burgers vector 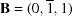 showing the edge part on the plane 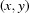 and the screw part along *z*. (*b*) The same object analysed as a two-dimensional tiling is a partial dislocation bounded by a planar defect of vertically oriented rhombi; as shown in (*a*), this defect is a dislocation of the 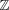-module generated by the projection of the three-dimensional simple cubic lattice onto the 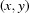 plane: it is a *module dislocation*. (*c*) Generating a similar module dislocation but from the cut of a four-dimensional hypercubic crystal makes the area’s overall relief much more difficult to grasp.

To give a first idea of what this question is about, let us consider the example shown in Fig. 1 ▸. At a first glance, it represents a slice in the 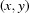 plane of a simple cubic lattice of a standard three-dimensional dislocation of Burgers vector 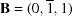 aligned along the *z* direction. Whereas the edge part of the dislocation is clearly seen in the 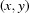 plane, the screw part along the *z* direction generates the one step height shaded in light grey. The drawing Fig. 1 ▸(*a*) is immediately understandable because of our natural spontaneous sense of visualizing three dimensions. But, if we consider this drawing for what it really is – in fact a simple two-dimensional tiling in the plane – then this same defect shown in Fig. 1 ▸(*b*) is less obvious: it is a partial edge dislocation of the two-dimensional periodic tiling bounding a row of reconstructed tiles – here rhombi rotated by 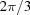 – that form a stacking fault line. This is now a partial dislocation in the two-dimensional subspace.

This example is quite trivial because the implied 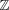-module has rank 3 but it becomes significantly more cumbersome to decipher defects based on 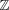-modules of higher rank where we lose our intuitive vision in 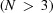-D space as illustrated in Fig. 1 ▸(*c*). We shall designate this kind of defect a * module dislocation* as opposed to the usual *lattice dislocation* to emphasize the fact that its Burgers vector belongs to the 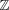-module and not to the lattice.

In §2, we briefly recall the tools we need to build a coherent crystallographic description of alloys having atoms located on a 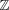-module, that we designate here as *module-based alloys*. These include:

(*a*) the well known cut-and-project method used to generate uniformly discrete sets of points that are quasiperiodic decorations of high-symmetry 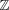-modules;

(*b*) the perpendicular shear technique that allows one to generate periodic approximants from these high-symmetry quasicrystals (Jarić & Mohanty, 1987 ▸; Gratias *et al.*, 1995 ▸).

In §3, we discuss the nature of the defects that can be generated while keeping the 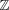-module invariant. These are:

(*a*) twins as discussed by Quiquandon *et al.* (2016 ▸);

(*b*) translation boundaries characterized by fault vectors 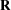 having irrational coordinates with respect to the unit-cell reference frame;

(*c*) *module dislocations* including those astonishing *metadislocations* found in specific approximants of i-AlPdMn icosahedral quasicrystals [see, for instance, Feuerbacher (2005 ▸) and Feuerbacher & Heggen (2010 ▸)] and the defects observed in approximants of the d-AlCuMn decagonal phase (Wang *et al.*, 2016 ▸);

(*d*) original, new kinds of dislocations with Burgers vectors having a zero component in the physical space, thus generating no displacement field and having no interactions with other dislocations and external stress fields; we call them *scalar dislocations*.

The last section of the paper summarizes our main conclusions.

## 2. *N*-D description of module-based alloys   

As already mentioned, several intermetallic periodic phases have structures with atoms located on a fraction of the sites of a 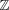-module. This happens each time the motif is made of atomic clusters with non-crystallographic symmetries, coherently interconnected and parallel to each other. Similarly to quasicrystals, these structures can be described as *rational* cuts of abstract periodic objects in spaces of dimension 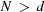. Describing and generating these module-based alloys require a few ingredients that are discussed next.

### Rank of the 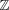-module   

2.1.

The first ingredient is the rank *N* of the 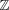-module as determined from the internal symmetry of the atomic cluster forming the motif. In the easiest cases, this rank is directly given by simple examination of the local symmetry of the motif when it has a point symmetry higher than that of the lattice of the crystal. For example, the rank 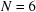 is quickly found for the many intermetallic phases that are approximants of icosahedral quasicrystals because their main atomic motifs are high-symmetry clusters, the atoms of which can all be indexed as integer linear combinations of the six unit vectors defined by the six quinary axes of the regular icosahedron.

**Figure 2:**
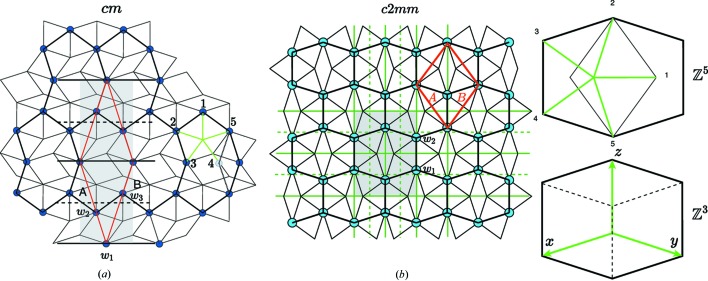
Examples of 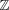-module models based on the module generated by the regular pentagon. (*a*) This structure (dark blue atoms) is a periodic ordered decoration (group *cm*) of the well known Penrose tiling built with the two golden rhombi with acute angles of 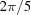 and 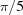 drawn in light grey. It is a substructure of the famous tiling originally drawn by Dürer (1525 ▸) built with two adjacent regular pentagons sharing an edge. (*b*) This honeycomb-like network of atoms (in light blue) with group 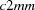 is a set of connected hexagons that are obtained by superimposing two opposite regular pentagons sharing a diagonal as shown on the right of the figure. The structure is described using the five-dimensional module of the regular pentagon but this same structure can also be viewed as the projection of a set of cubes, and thus be described by the three-dimensional projection of the cube.

For illustrating our purpose, we shall use here two two-dimensional examples that can be analysed as two-dimensional periodic (low) approximants of the famous Penrose tiling (Penrose, 1979 ▸) built with the two golden rhombi of acute angles 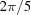 and 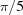. Here, the natural dimension of the *N*-D lattice Λ is 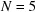 corresponding to the 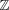-module generated by the regular pentagon.^2^ Such is the case of the well known Dürer structure (Dürer, 1525 ▸) made of a periodic arrangement of adjacent pentagons sharing an edge. To make our toy model example a little more original, we remove one vertex of the pentagon, getting then a *bean* structure as shown in Fig. 2 ▸. In the five-dimensional frame, this structure has a lattice 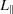 with a primitive unit cell defined by 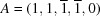, 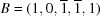 with three translation orbits^3^ 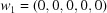, 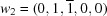 and 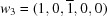. The Dürer structure is obtained by adding the fourth translation orbit 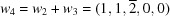.

In some other cases, the determination of the rank of the module is not so obvious.

Indeed, our second example shown in Fig. 2 ▸(*b*) is a honeycomb network built with hexagons defined by the superimposition of two regular opposite pentagons sharing a diagonal as shown in the top right of Fig. 2 ▸(*b*): the lengths of the segments 2–5 and 3–4 are in the ratio of the golden mean 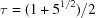 and all vertices in blue in the structure of Fig. 2 ▸(*b*) can be labelled as linear integer sums of the five unit vectors of the regular pentagon. Here again, we can choose the natural 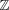-module of the regular pentagon and define the atomic structure in five-dimensional space by the primitive unit cell 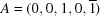 and 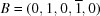 with two translation orbits 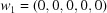 and 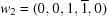 (see Fig. 2 ▸). But because this tiling is made of hexagons that can always be seen as convex envelopes of the two-dimensional projection of cubes, the structure can also be viewed as belonging to a 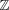-module of rank 3 (instead of 5) as seen in the bottom right of Fig. 2 ▸. In that case, the three-dimensional unit cell is now defined by 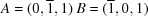 with translation orbits 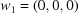, 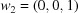. The connection with the five-dimensional description is given by expressing the basic three-dimensional unit vectors in terms of those of the five-dimensional basis: 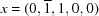, 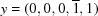, 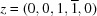. Choosing either 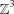 or 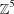 depends on which defect is studied: a simple dislocation can be described using 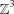 whereas a 5-f twin can be generated only on the basis of 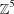. This point will be exemplified later.

### 2.2. The cut method   

**Figure 3:**
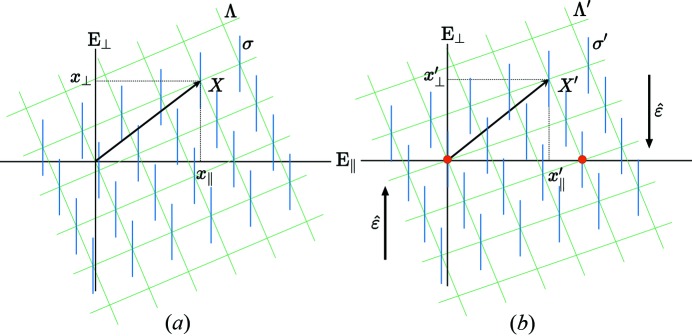
(*a*) Generating a uniformly discrete set out of a dense 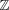-module resulting from a *d*-dimensional projection in 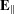 of an *N*-dimensional lattice Λ consists of attaching to each *N*-D lattice node of Λ a (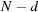)-D bounded volume σ parallel to 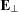 designated here as an atomic surface (AS) and collecting the intersection points of these ASs with 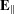. (*b*) To generate a periodic structure based on the same 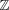-module, a shear along 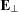 is applied that brings specific nodes of Λ parallel to 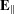. These nodes define the lattice 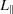 of the periodic structure in 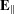.

Once the rank of the module has been determined, the next step consists of generating the structure itself that is a *long-range-ordered set* of points out of the 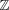-module. We use here the well known cut-and-project method initially derived to describe quasiperiodic structures (see Fig. 3 ▸). It consists of projecting an *N*-D lattice Λ in a *d*-D subspace (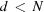) in a direction that is irrational with the *N* periods of Λ. Because the projection 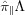 is a dense set of points, an additional criterion is used in the complementary subspace 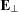 that consists of selecting only those lattice points of Λ that project in 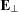 inside a given finite bounded (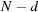)-D volume 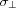 that we designate as an *atomic surface* (AS). This generates a uniformly discrete set of points 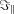 that is a subset of the 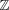-module 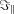: 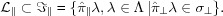



### 2.3. The perpendicular shear method   

To generate subsequently a *periodic* structure, we apply a shear of the *N*-D lattice Λ along 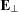 – thus keeping the original module in 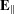 invariant – in order to align *d* chosen independent nodes of Λ along 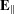 by the transformation (Gratias *et al.*, 1995 ▸; Quiquandon *et al.*, 1999 ▸): 
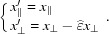
This generates a *d*-D lattice 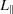 in 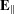. Let 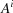 be the *d* vectors of Λ, the projections of which in 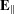 define the unit cell of the structure. To ensure the generated structure is periodic of periods [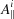] the shear matrix 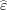 must be such that 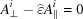
and therefore 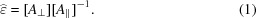
This technique of imposing a perpendicular shift of Λ is very efficient: it allows one to generate infinitely many periodic structures all based on the same 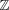-module.

### 2.4. The atomic surfaces   

ASs are among the most important concepts in the description of (perfect) quasicrystals since they define the densities and relative locations of the atomic species of the structure. A quasicrystalline structure is defined by specifying for each chemical species the complete collection of ASs (bounded polyhedra in the case of icosahedral phases) and their relative locations in the *N*-D space. The real structure in 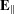 is thus generated by the cut algorithm. Depending on where the cut is performed along 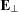, the structures obtained differ from each other. If the projection of Λ is dense everywhere in 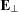, these structures form a dense enumerable set of locally isomorphic and physically indistinguishable structures related to each other by *phasons* (local retilings) that are analysed as local fluctuations of 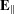 in 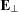.

Deriving ASs for the case of periodic structures is the unique conceptual difficulty in our present approach. Indeed, because the final projection leads to a periodic structure in 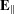, the notion of AS loses *a priori* physical pertinence since the projection of the *N*-D lattice in 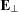 is now a lattice, say 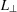, *i.e.* a *discrete set* of points instead of being a *dense set* as in the quasicrystalline case. This obliterates the basic one-to-one relation in quasicrystals between the projections of the nodes of the *N*-D lattice Λ in 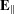 and those in 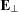. In the periodic case, each projection in 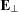 of a node of Λ is now associated with an *infinite set of sites* in 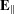, made of all the equivalent positions deduced from each other by the lattice 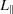 of the structure. These sets are the *translation orbits* that we introduced in the preceding section. Translation orbits are the objects that restore the one-to-one correspondence between 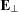 and 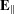: to each lattice node in 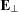 is associated one and only one translation orbit in 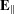 and *vice versa*. This reduces the physical significance of an arbitrary displacement of the cut in 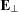 to the only case where this displacement is a translation of 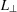.

**Figure 4:**
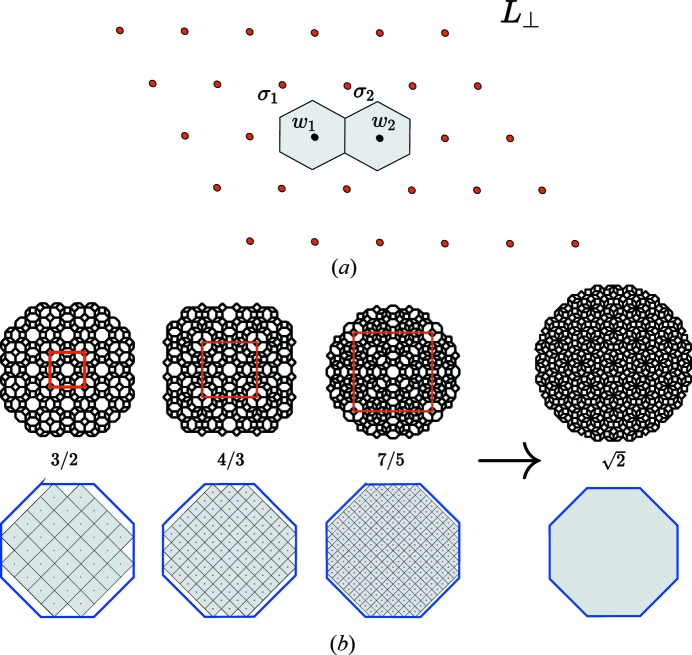
(*a*) Typical two-dimensional example of defining the ASs of a (periodic) structure with two translational orbits 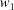 and 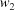 represented in 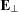 with projected lattice 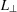: the ASs are formed by the union of the two Voronoi cells 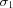 and 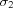 (in grey) centred on each of the translation orbits. (*b*) The union of the Voronoi cells (in light grey) of successive approximants of the octagonal tiling compared with the usual ASs defined by the convex envelopes (in blue) of the four-dimensional unit cell: as the order of the approximant increases the union of the Voronoi cells tends towards the canonical AS of the octagonal tiling.

It is however very useful to keep the concept of ASs alive in the case of periodic structures in order to possibly compare the structural properties of both periodic and quasiperiodic structures using the same cut-and-project method in a unified way. In fact, for the periodic case, any AS is acceptable if it satisfies the condition that, up to a global translation in 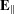, the atomic structure generated by the cut is unique and thus independent of the choice of the trace of the cut in 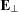. This means that the union of the projections in 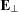 of identical ASs forms a covering of 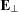 such that no space is left (localizing the cut there would give no structure at all) and no overlap appears (there would be at least two different structures generated depending on where the cut passes in 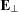, in an overlap region or not). This set must therefore be a tiling of 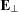. The simplest way to meet this requirement of using identical cells that form a tiling of 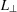 is to define the ASs in 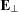 as the *union of the half-opened* 4 *Voronoi cells centred at the nodes of* 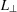 *associated with the translation orbits* of the structure as illustrated in Fig. 4 ▸.

This definition is not only the most natural but it presents the advantage of leading to the usual geometry of quasicrystals when applied on a series of convergent approximant structures as shown in Fig. 4 ▸(*b*). Here, each higher-order periodic approximant of the octagonal phase is described by an increasing number of translation orbits distributed on the nodes of a denser lattice 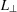 with smaller Voronoi cells. At the infinite limit, the union of the half-opened Voronoi cells superimposes on the standard canonical ASs used in the standard tiling theory of quasicrystals.

The immediate consequence of the present definition of ASs for periodic structures is that it obliterates the possible existence of the so-called *phasons* typical of quasicrystals and incommensurate phases: here, any crossing of the AS boundaries in 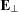 leads in 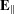 to either no change at all, or to a global translation of the same structure. This can be particularly well understood by examining the approximant structures of the octagonal tiling shown in Fig. 4 ▸(*b*): the empty sites in the successive approximants are the positions of easy tile flips, *i.e.* phason sites.

## 3. Generating module defects   

Defining defects in solids requires one first to define what is chosen as the reference for ideal perfect structures. Here, the basic reference is the 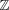-module in 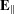 that is the projection of the *N*-D lattice Λ. Thus the reference object is Λ, the symmetry group 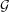 of which is the set of the isometries 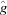 of the *N*-D space that leave both Λ and 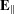 invariant, *i.e.* those isometries 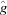 that commute with the projector 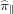: 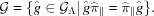
This group 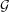 is a supergroup of the group 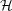 of the actual structure in 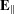 and the decomposition of 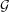 in cosets of 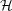, 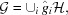
defines all the possible defects of the real structure that leave the 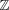-module invariant.

Because 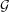 has the lattice Λ in the *N*-D space as translation subgroup whereas 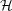 has the lattice 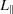 in a *d*-D subspace, the number of translational cosets is infinite^5^ and an additional criterion – discussed later – is necessary to select those specific translational boundaries that can plausibly exist between adjacent variants in 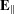.

The orientational defects, in contrast, are issued from the coset decomposition of the point groups that lead to a finite number of variants. These defects are twins that we can qualify as merohedral in the sense of Friedel (1904 ▸, 1926 ▸, 1933 ▸) where the notion of lattice is replaced by that of 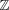-module (Quiquandon *et al.*, 2016 ▸).

### 3.1. Explicit examples   

Let us consider our two previous examples shown in Fig. 2 ▸. They both are subsets of the 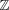-module generated by the regular pentagon projection of a five-dimensional lattice in the configurational five-dimensional Euclidean space that decomposes according to 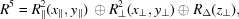
where 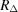 is an overabundant dimension, the rational one-dimensional line along the main diagonal 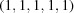.

Starting from a five-dimensional node 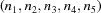, we obtain its components using the usual formulas (see, for instance, Duneau & Katz, 1985 ▸): 
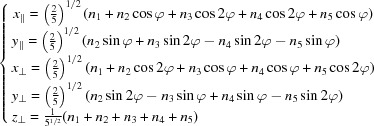
where 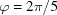. Introducing the golden mean 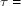 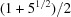 and observing that 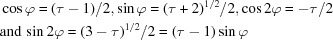
we can write these relations in a compact form: 
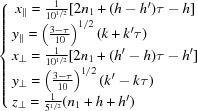
using the variables 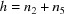, 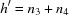, 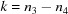, 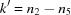 similar to those introduced in the indexing scheme of the icosahedral quasicrystalline phases (Cahn *et al.*, 1986 ▸). We note that 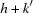 and 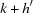 are even numbers and the transformation from 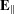 to 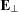 consists of applying the following simple substitution rules: 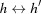 and 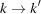, 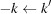.

**Figure 5:**
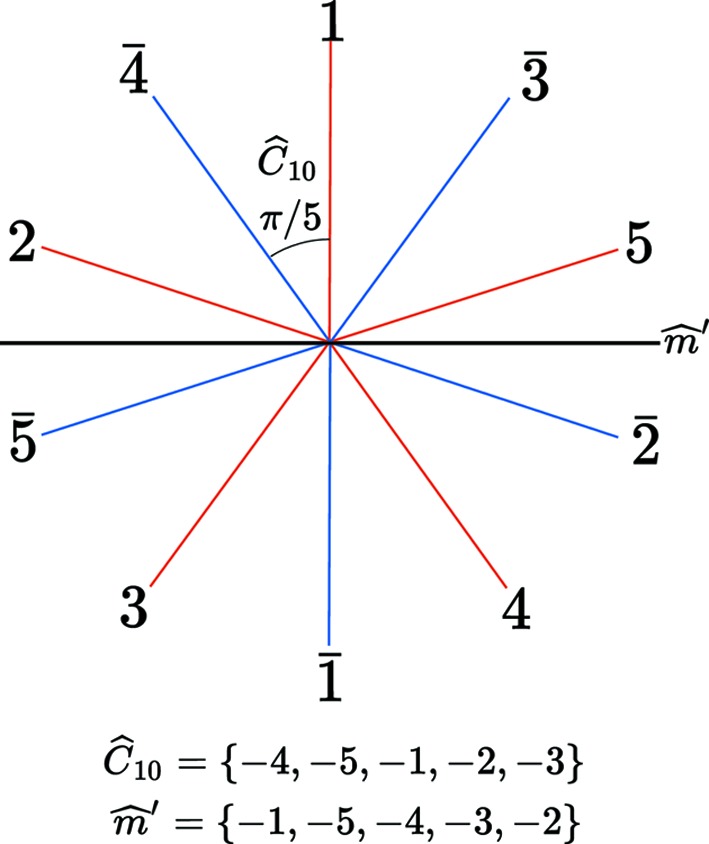
Generating the point group 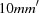 requires two generators: the rotation 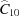 of angle 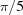 and the mirror 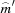. This point group has 20 elements corresponding to the symmetry of the regular decagon. It is the intrinsic symmetry group of the five-dimensional lattice that keeps the physical space 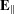 invariant.

The total symmetry group of the five-dimensional hypercubic lattice has 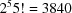 elements but only the subgroup 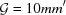 with 20 elements leaves 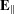 invariant. This point group is generated by the rotation 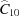 of 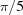 and the mirror 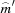 as drawn in Fig. 5 ▸. An economical way of writing symmetry operations is by using signed permutations. For example, the mirror 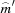 defined in Fig. 5 ▸ tranforms 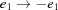, 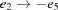, 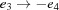, 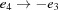, 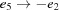 or in matrix form: 
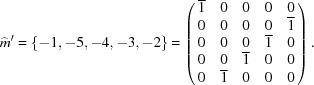



#### 3.1.1. The bean structure   

The primitive unit cell of the bean structure is defined by the two five-dimensional vectors 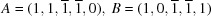, both perpendicular to 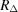 with three translation orbits 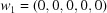, 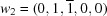 and 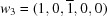. The two-dimensional lattice 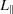 is defined by 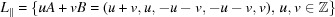
projecting in 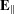 as 
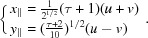
The shear matrix 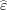 reduces thus to a 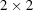 matrix connecting 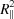 with 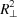, the one-dimensional subspace Δ being invariant under the shear. Using equation (1), we obtain after a few algebraic calculations 
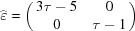
leading to 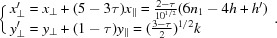
The projected lattice in 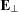, 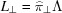, is generated by the three vectors 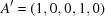, 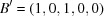 and 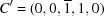: 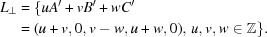



#### 3.1.2. The honeycomb structure   

The unit cell of the honeycomb structure is defined by the two five-dimensional vectors 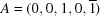 and 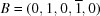, both per­pendicular to 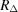 and with two translation orbits 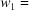 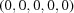 and 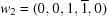. The two-dimensional lattice 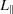 is defined by 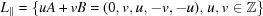
projecting in 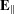 as 
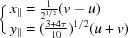
and the shear matrix is 
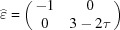
leading to 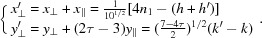
The projected lattice in 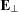, 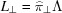, is generated by the three vectors 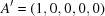, 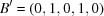 and 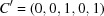: 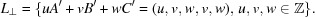



### 3.2. Twins   

Twin operations in the present context are orientational defects between variants that share the same 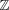-module. In a previous paper (Quiquandon *et al.*, 2016 ▸), we proposed calling them merohedral twins after Georges Friedel (Friedel, 1926 ▸) by extending the role of the lattice to the 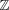-module.

**Figure 6:**
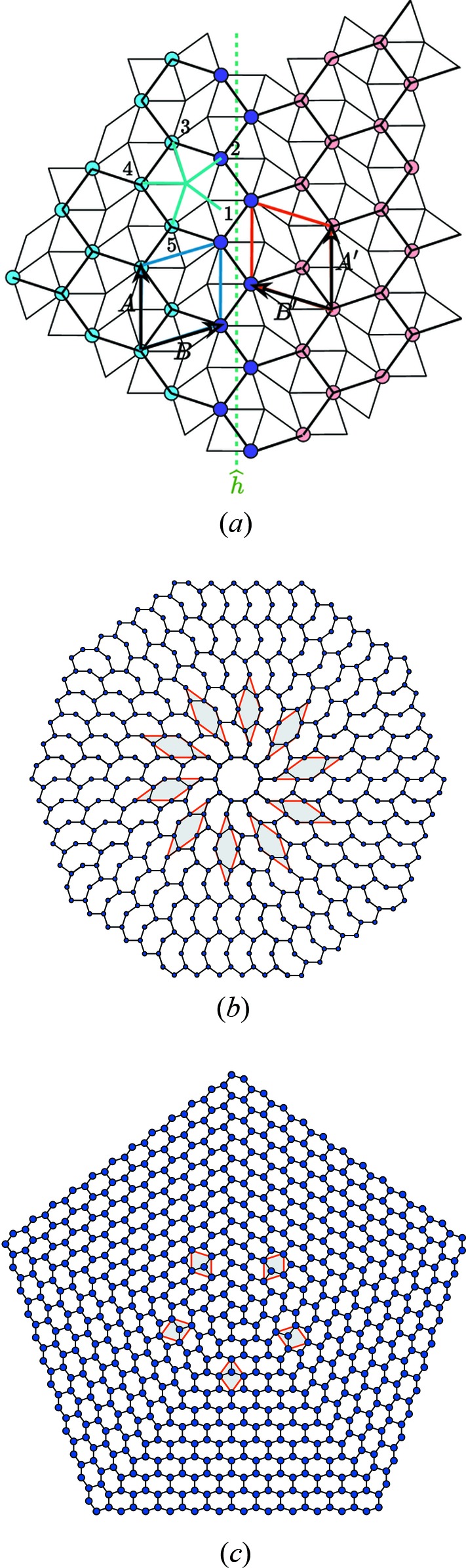
(*a*) Coherent *merohedral* twin of the honeycomb structure: the twin operation 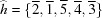 is a mirror with an irreducible translation part 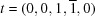; it transforms the unit cell {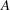 = 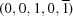, 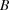 = 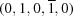} into 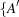 = 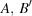 =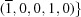. This interface is perfectly coherent with two rows of common atoms (drawn in purple) and is based on the elementary rhombi of the Penrose tiling drawn in thin lines. (*b*), (*c*) The twin variants generated by the decomposition of 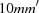 on (*b*) 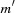 (bean structure) with 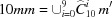 and on (*c*) 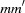 (honeycomb structure) with 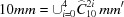. As can be clearly seen here, all interfaces are perfectly coherent although there is no two-dimensional coincidence lattice between any two adjacent twin individuals.

An example of such merohedral twins in the honeycomb structure is shown in Fig. 6 ▸(*a*). It is defined by the mirror operation 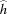 that belongs to the symmetry group 10*mm* of Λ: 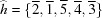 associated with the translation 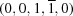. This symmetry operation does not survive under projection on 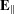: it generates a coherent twin equivalent to a rotation by 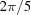 as illustrated in Fig. 6 ▸(*c*) where the coset decomposition of 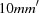 on 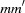 gives five variants. As required, all twin individuals are built on the *same* module, thus justifying the term of *merohedral* twins. Concerning the bean structure, the coset decomposition of 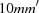 on 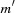 gives ten variants shown in Fig. 6 ▸(*b*). Here, again, all ten variants share the same and unique 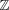-module.

### 3.3. Translation defects   

**Figure 7:**
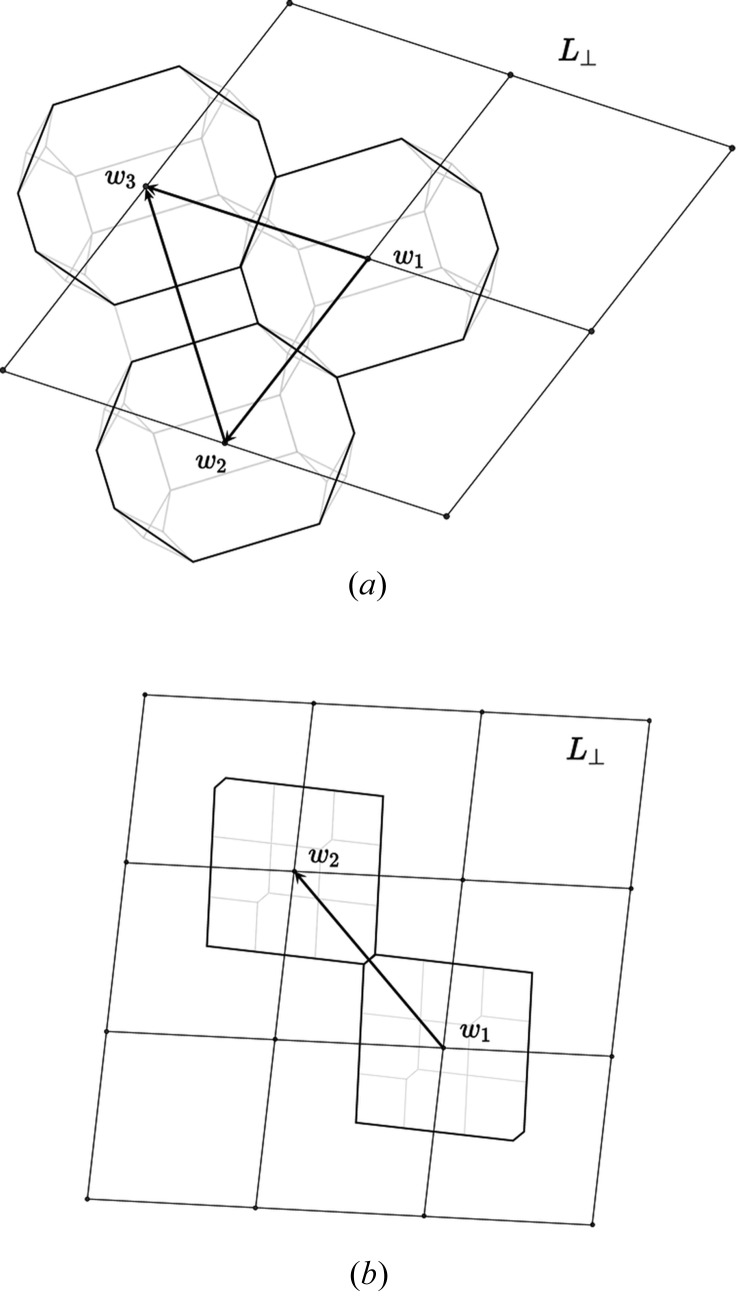
(*a*) The bean structure represented in 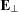 is generated by three Voronoi cells located at 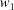 = 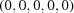, 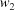 = 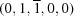 and 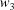 = 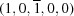; there are thus three most plausible translation boundaries 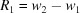, 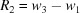 and 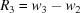. (*b*) The honeycomb structure represented in 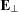 is generated by two Voronoi cells located at 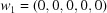 and 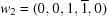. Its most plausible translational defect is thus the boundary characterized by 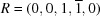 that leaves one translational orbit invariant (see Fig. 8 ▸).

As previously mentioned, the translation defects are issued from the coset decomposition of Λ onto 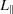 and are thus infinitely many. For predicting which translation boundaries are plausibly expected to occur, we need an additional geometrical criterion. A reasonable choice is to search for a maximum continuity between adjacent translational variants, *i.e.* maximizing the overlap between the atomic orbits of variants. This is easily achieved by considering the structure in 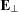, *i.e.* a set of Voronoi cells attached to a finite collection of nodes 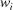 of the lattice 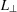, each 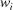 corresponding to a translational orbit in 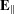 (see Fig. 7 ▸).

**Figure 8:**
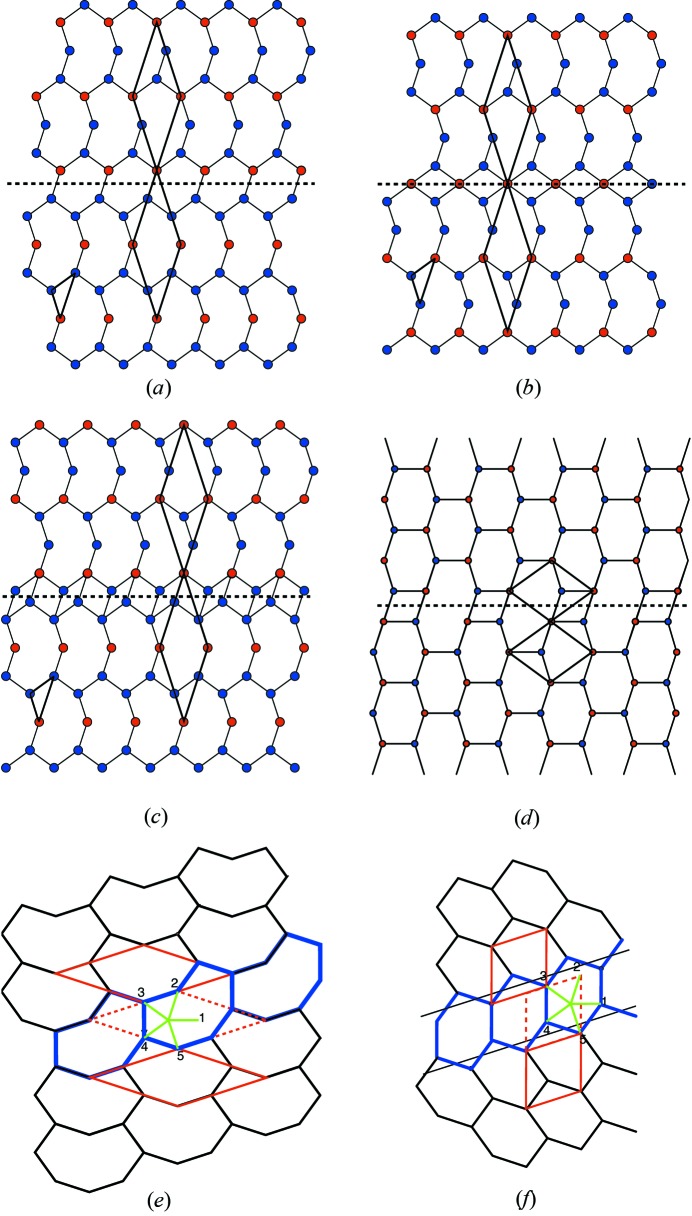
The translation boundaries of the bean structure associated with (*a*) 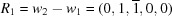, (*b*) 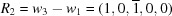, (*c*) 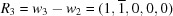; in all three cases, one (in red) over the three translation orbits is invariant on crossing the boundary. (*d*) The unique translation boundary of the honeycomb structure 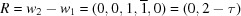. See Fig. 7 ▸ for the references of the translation orbits in 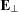. (*e*)–(*f*) Example of the translation 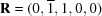 that can be achieved by introducing a microtwin: the microtwin is realized by successive application of a twin operation and its inverse displaced by 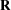: on (*e*) it is a rotation *h* of 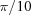 followed by its opposite 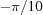 and on (*f*) it is a mirror applied twice.

Our strategy is thus to choose those translations 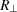 of 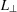 that superimpose a maximum number of Voronoi cells on top of each other in order to generate adjacent variants sharing a maximum number of translational orbits. For example, since the honeycomb structure is defined with two translation orbits 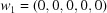 and 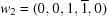, the only translation boundary we can expect that leaves one orbit invariant is the boundary generated by the fault vector 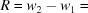 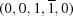, as shown in Fig. 8 ▸(*d*).

The case of the bean structure is slightly more complicated since it is generated by three Voronoi cells. This offers then three possible fault vectors 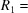 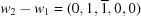, 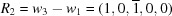 and 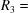 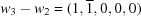, each leaving one translation orbit invariant among the three of the structure as depicted in Figs. 8 ▸(*a*), 8 ▸(*b*) and 8 ▸(*c*).

Another way of generating simple translation defects consists of using fine slabs of twinned variants inside a main crystal (microtwins). This is achieved by applying a twin operation as discussed in the previous subsection, say 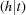, and, subsequently, its inverse displaced by a lattice translation 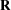 of Λ, 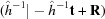, leading to 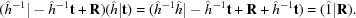
This is exemplified in Figs. 8 ▸(*e*) and 8 ▸(*f*). Successive introductions of *n* such elementary slabs generate global translations of 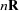 between the two parts of the original crystal.

### 3.4. Module dislocations   

**Figure 9:**
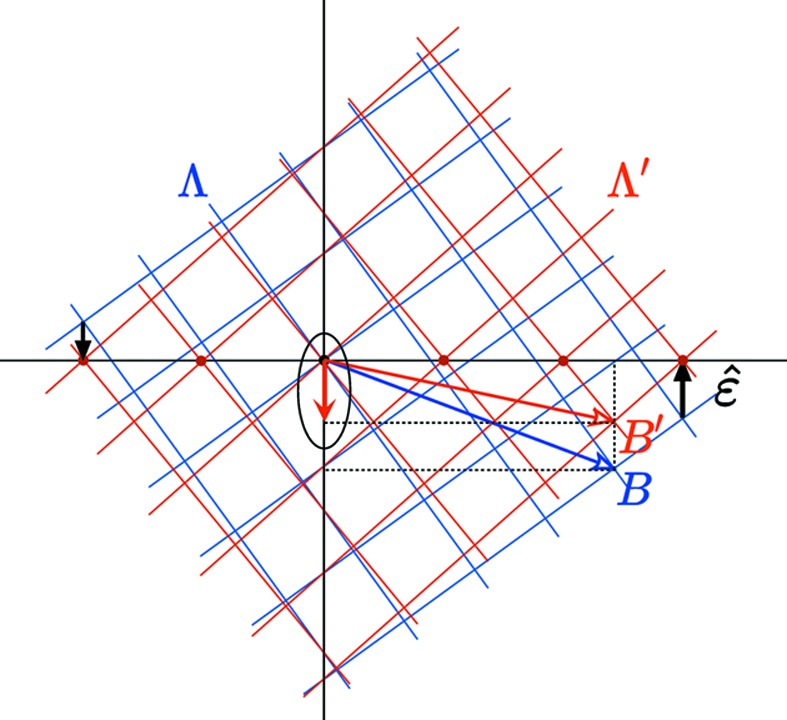
A 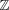-module dislocation is the image in 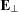 of a perfect dislocation of Λ in *N*-D space, of Burgers vector 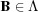 that has a *non-zero component* 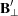 in 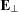 *after the shear* 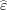.

The previous translation boundaries with fault vectors 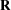 belonging to the 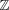-module can be bounded by partial dislocations of Burgers vectors 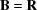. These module dislocations are defined as perfect dislocations of the lattice Λ, the Burgers vectors of which have a *non-zero component* in 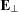 *after the shear* 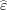 as illustrated in Fig. 9 ▸: 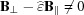
as opposed to usual dislocations for which 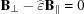.

They are the natural extensions for the approximants of the usual dislocations encountered in quasicrystals and correspond to the so-called *metadislocations* first observed by Klein *et al.* (1999 ▸); they were discussed by Klein & Feuerbacher (2003 ▸) from the the pioneering work by Beraha *et al.* (1997 ▸) and Klein *et al.* (1997 ▸) on the approximant structures 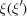-AlPdMn. These defects have been extensively and magnificently studied using high-angle annular dark-field (HAADF) electron microscopy by Feuerbacher and co-workers (see, for instance, Heggen *et al.*, 2008 ▸; Feuerbacher *et al.*, 2008 ▸; Feuerbacher & Heggen, 2010 ▸). Recent analogous, superb observations have been made by Wang *et al.* (2016 ▸) on approximants of the decagonal phase of the AlCuMn system. All these observations testify to the fact that the observed defects are indeed geometrically connected to an underlying tiling but none offers a general framework able to properly define what they really are. The connection to an *N*-D description has been clearly demonstrated by Engel & Trebin (2006 ▸) on the basis of the experimental observations of Feuerbacher and co-workers. A first general attempt to define metadislocations in the *N*-D framework has been proposed by Gratias *et al.* (2013 ▸). Finally, in the present paper, we wish to definitely emphasize the fundamental *N*-D character of these defects in designating them by the accurate name of *module dislocation* rather than *metadislocation*, which is not very informative.

These module dislocations differ from usual dislocations in crystals in two basic ways:

(i) the Burgers vector 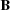 is a vector of Λ in *N*-D space so that the 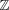-module is left invariant by the dislocation;

(ii) since the Burgers vector 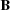 has a non-zero component in 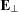 after shear, the dislocation is a partial dislocation bounded by one or several stacking fault boundaries.

**Figure 10:**
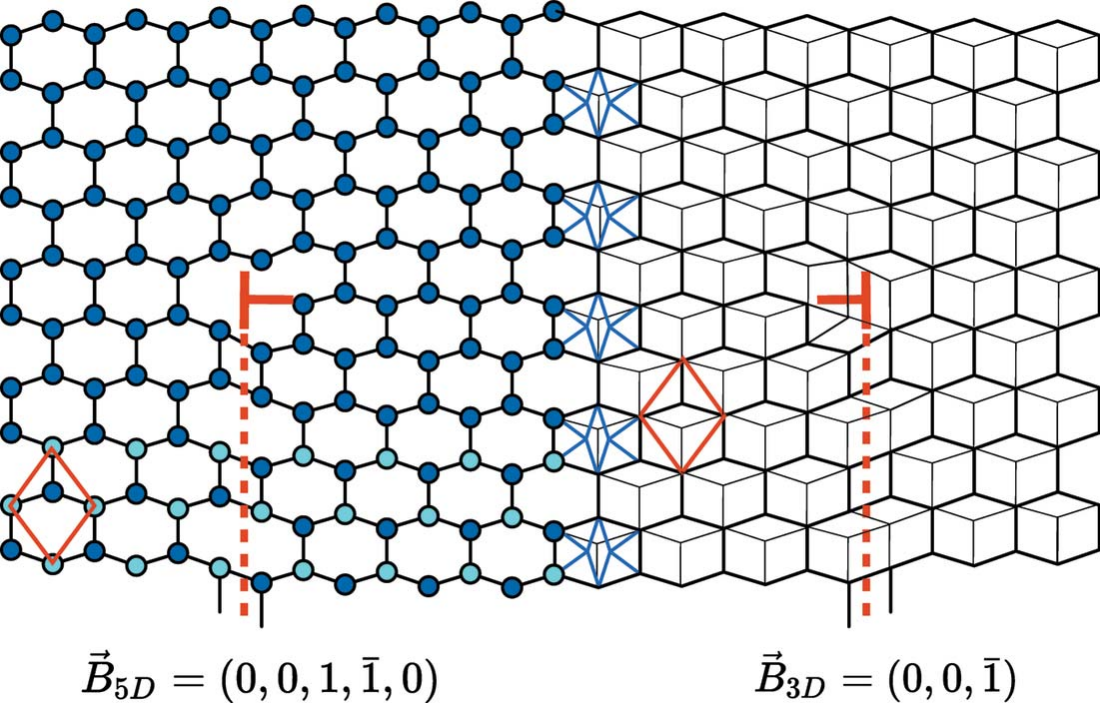
A typical 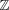-module dislocation dipole in the honeycomb structure that illustrates the five-dimensional lattice Λ description with Burgers vector 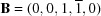 on the left and the three-dimensional lattice with Burgers vector 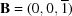 on the right. Of course, both descriptions are totally equivalent.

This is exemplified in Fig. 10 ▸ with a simple dislocation 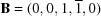 of the five-dimensional representation on the left, or equivalently by 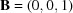 in the three-dimensional representation on the right. This last representation clearly shows the three-dimensional nature of the dislocation and its associated stacking fault.

#### 3.4.1. Scalar dislocations   

There is a special situation that arises when using an overdetermined 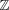-module, *i.e.* when 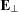 contains one or more rational directions of the lattice Λ. Such is the case in our two previous examples based on the regular pentagon described in five dimensions with the introduction of the additional one-dimensional periodic subspace 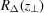 in 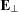.

There, particular dislocations may be found that have a non-zero Burgers vector in Λ but that have a zero 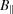 component in the physical space. Those strange dislocations have the remarkable property of generating no defomation field and thus of being insensitive to any stress fields and to any other dislocations. This is easily understandable in terms of tilings in which the topological fault introduced by the dislocation is fully accommodated by a simple retiling of the elementary protiles with no deformation. We therefore propose designating this special kind of topological defect as a scalar dislocation since its main characteristic is the length of the Burgers vector – a scalar property – and not the vector by itself.

**Figure 11:**
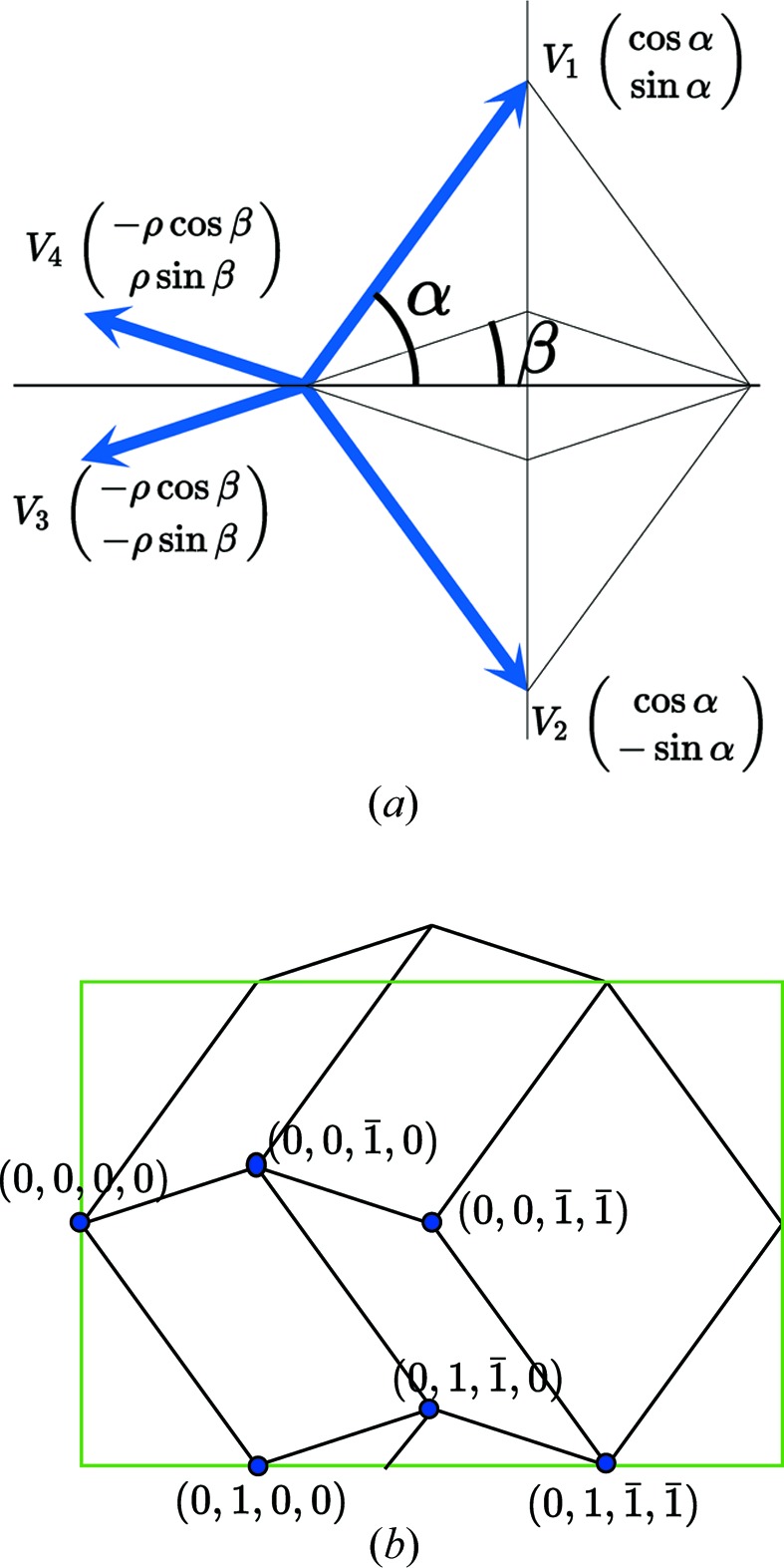
Scalar dislocation of Burgers vector 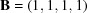 in a tiling described from a four-dimensional space with an overdetermined module where the four basic vectors have their projections in 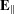 summing up to zero, 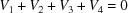, as shown in (*a*). The periodic structure is seen in (*b*); it has lattice parameters 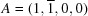 and 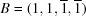 and is generated by six translation orbits.

To exemplify this intriguing situation, we consider the two-dimensional structure shown in Fig. 11 ▸ built with the four vectors 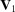, 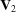, 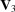 and 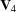 such that 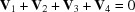. The configurational four-dimensional Euclidean space decomposes as 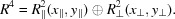
Using the coordinates of the four vectors in 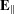, 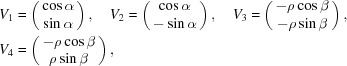
we note that 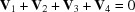 imposes 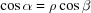 and 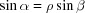.

**Figure 12:**
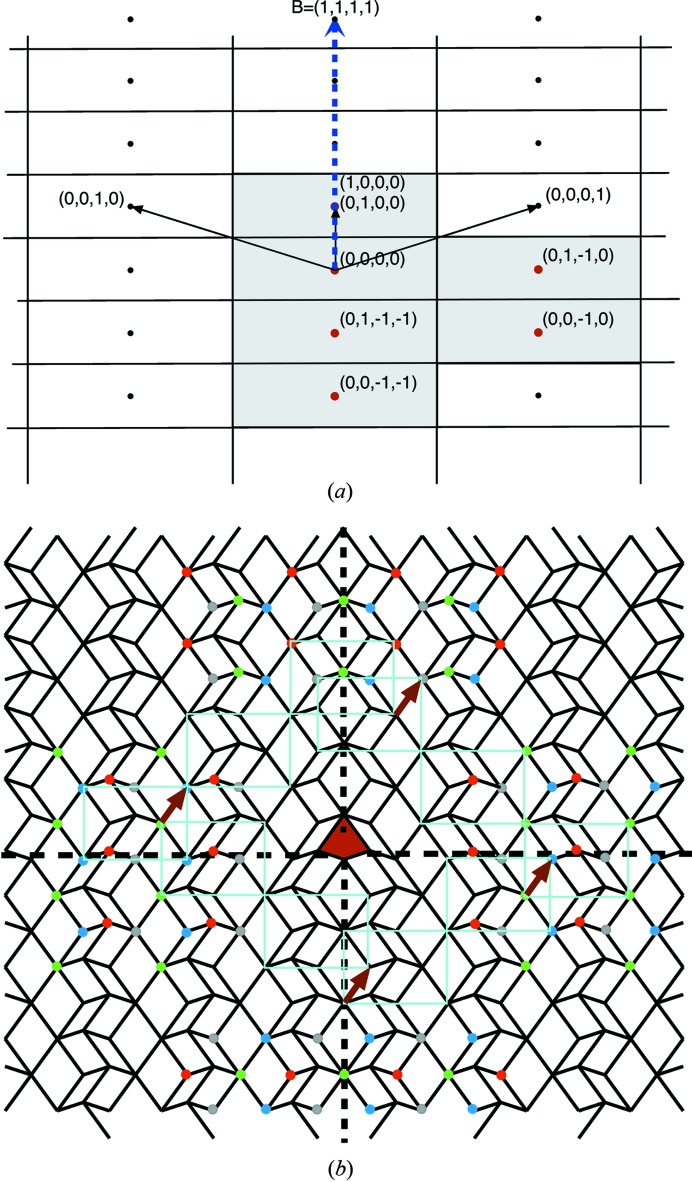
(*a*) The structure of Fig. 7 ▸(*b*) projected in 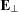 is defined by the atomic surface union of the six Voronoi cells in grey located at the projections in 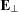 of the six translation orbits. (*b*) The Burgers vector 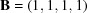 is contained in 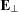 and thus generates no deformation of the tiles, whatever their location in the physical space as shown here. The defect (in red) is at the intersection of four translation boundaries, each conserving four among the six of the orbits forming the structure.

Let (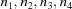) be a node of the four-dimensional lattice Λ, (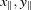) and (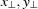) its components in, respectively, 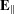 and 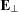. Simple algebraic manipulations lead to the following transformation rules normalized by the global scale factor 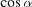: 
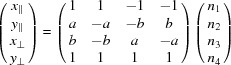
with 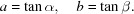
Thus, the basic parent quasiperiodic structure is one-dimensional quasiperiodic along 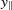 – according to the relative values of the angles α and β – and periodic along 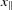 with one-dimensional unit-cell parameter 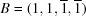. Correlatively, the perpendicular projection is dense along the 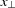 direction and periodic along the 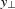 direction with period 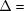 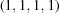: 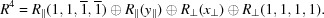
To obtain the actual periodic structure with a two-dimensional unit cell defined by 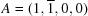 and 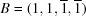 we apply a shear along 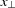 proportional to 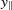, thus reducing the 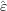 matrix to a simple number: 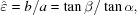
leading to 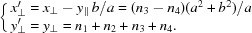
The structure is defined by six translation orbits shown in Fig. 11 ▸(*b*), 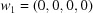, 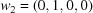, 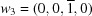, 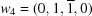 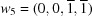 and 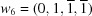 with the lattice 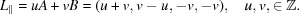
Introducing the dislocation of Burgers vector 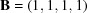 that has a zero component in 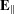 leads to a point defect shown in red in Fig. 12 ▸ that is bounded by four lines of translation faults. Because the dislocation induces no deformation, the four fault vectors 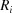 are defined up to any translation of the lattice as depicted in Fig. 12 ▸, the global geometrical consistency being
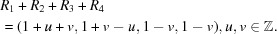
A simple solution proposed in Fig. 12 ▸, heavy dark red arrows, is to choose 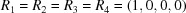 leading to 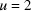 and 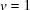 in the previous expression. This shows that each boundary is associated to move the six Voronoi cells along the projection of the 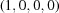 direction, that is 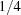 in length of the 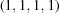 direction. This move keeps four of six invariant Voronoi cells and therefore four translation orbits are invariant out of the six forming the structure on each crossing of the translation boundaries (see Fig. 12 ▸). This makes these boundaries remarkably coherent: all are made of a local coherent redistribution of the original tiles with no additional new external shapes.

## 4. Conclusion   

We have seen that those alloys for which the atoms are long-range ordered on a non-trivial 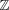-module, in addition to being periodically spaced, can contain new original defects corresponding to internal symmetry operations of the 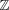-module that are lost because of the periodicity. These defects are twins, translation defects and dislocations that we call module dislocations to differentiate them from standard lattice dislocations, and appear as partial dislocations bounded by one of several translation faults. We have seen that for the case of overdetermined modules specific dislocations can exist with Burgers vectors having a zero component in the physical space. These dislocations, which we call scalar dislocations, are located at the intersection of translation defects and are well described by a collection of local retilings with no deformation of the prototiles.

## Footnotes

1
-modules are the natural extension of lattices. A -module of rank *N* in  with  is the set  of points in  such that   with  where the *N* vectors  are arithmetically independent (*i.e.* no non-zero integer combination of the *N* vectors gives the null vector): (i) any -module of rank *N* in  is the (irrational) projection of a lattice  in ; (ii) a -module of rank *N* in   forms an enumerable dense set of points in  or in a non-empty subspace of ; (iii) if  the -module is trivially a lattice .

2 This technique of using a five-dimensional hypercubic lattice instead of the usual four-dimensional root lattice makes the pentagonal symmetry explicit and all algebraic manipulations much easier; it is similar to using four indices in the hexagonal crystalline system.

3 The translation orbit  is the set of the equivalent points of  generated by the translations of :  irrespective of the point symmetry of the structure.

4 The Voronoi cells form the canonical tiling associated with the lattice : in the case where the cut passes at the boundary between two adjacent cells, a decision must be taken to choose one of the two cells; because Voronoi cells are always centred, we define the ASs as half-opened cells, *i.e.* that include a boundary and exclude its opposite, like the segment  for the one-dimensional case.

5 This corresponds to the fact that in  the lattice of the periodic structure defines a discrete set of points whereas the -module defines a dense set of points.
